# Sprachverstehen in Abhängigkeit von der cochleären Abdeckung – Vergleich bei bimodal versorgten Cochleaimplantatpatienten

**DOI:** 10.1007/s00106-023-01330-w

**Published:** 2023-07-14

**Authors:** Tobias Rader, Leonhard Schrank, Jennifer L. Spiegel, Pascal Nachtigäller, Judith E. Spiro, John-Martin Hempel, Martin Canis, Joachim Müller

**Affiliations:** 1 Abteilung Audiologie, Klinik und Poliklinik für Hals-Nasen-Ohrenheilkunde, LMU Klinikum der Universität München, Marchioninistr. 15, 81377 München, Deutschland; 2 Klinik und Poliklinik für Hals-Nasen-Ohrenheilkunde, LMU Klinikum der Universität München, München, Deutschland; 3 Deutsches Schwindel- und Gleichgewichtszentrum, LMU Klinikum der Universität München, München, Deutschland; 4 Klinik und Poliklinik für Radiologie, LMU Klinikum der Universität München, München, Deutschland

**Keywords:** Hörgeräte, Prothesen und Implantate, Computertomographie, Insertionstiefe, Sprachaudiometrie, Hearing aids, Prostheses and implants, Computed tomography scan, Insertion depth, Speech audiometry

## Abstract

**Hintergrund:**

Der Hörerfolg für Patienten mit bimodaler Versorgung, bestehend aus einem Cochleaimplantat (CI) und einem kontralateralen Hörgerät (HG), ist unterschiedlich. Einige Patienten profitieren von einer bimodalen Versorgung mit CI und HG, andere nicht.

**Ziel der Arbeit:**

Ziel war die Untersuchung des Erfolgs beim Sprachverstehen bimodal versorgter Patienten in Abhängigkeit von der cochleären Abdeckung (Cochlear Coverage, CC) durch den CI-Elektrodenträger.

**Material und Methoden:**

Mittels der Software OTOPLAN (Fa. CAScination AG, Bern, Schweiz) wurde retrospektiv die CC von 39 Patienten anhand präoperativer Computertomographien (CT) des Felsenbeins erhoben, und in die Patienten wurden in 2 Gruppen, zum einen mit einer CC ≤ 65 % (CC^500^) und zum anderen mit einer CC > 65 % (CC^600^), unterteilt. Das monaurale Sprachverstehen für Einsilber bei 65 dB Schalldruckpegel (Sound Pressure Level, SPL) im Freifeld wurde zu verschiedenen Beobachtungszeitpunkten, präoperativ mit HG und postoperativ mit CI, erfasst und zwischen den Gruppen verglichen. Das Sprachverstehen wurde des Weiteren mit der CC der Gesamtkohorte präoperativ und zum Nachbeobachtungszeitpunkt (NBZ) korreliert.

**Ergebnisse:**

Es wurde kein signifikanter Unterschied im Sprachverstehen zwischen Patienten mit CC^500^ und Patienten mit CC^600^ zu den einzelnen Beobachtungszeitpunkten festgestellt. Sowohl bei Patienten mit CC^500^ als auch bei Patienten mit CC^600^ kam es zu einer stetigen Verbesserung des Sprachverstehens nach der Implantation. Während Patienten mit CC^600^ im Trend eher eine frühere Verbesserung des Sprachverstehens zeigten, wiesen Patienten mit CC^500^ tendenziell eine langsamere Verbesserung in den ersten 3 Monaten und danach eine steilere Lernkurve auf. Zum NBZ näherten sich die beiden Patientengruppen ohne signifikante Unterschiede im Sprachverstehen an. Es gab keine signifikante Korrelation zwischen dem unimodalen/unilateralen Sprachverstehen im Freifeld und der CC. Allerdings erreichten v. a. die Patienten, die eine CC von 70–75 % aufwiesen, das maximale Sprachverstehen.

**Schlussfolgerung:**

Trotz einer nichtsignifikanten Korrelation zwischen CC und Sprachverstehen schien v. a. die Patientengruppe mit einer Abdeckung von 70–75 % das Maximum im unimodalen/unilateralen Sprachverstehen zu erreichen. Es besteht jedoch Raum für weitere Untersuchungen, da CC^500^ mit einer kürzeren Cochlear Duct Length (CDL) assoziiert war und in beiden Gruppen lange und sehr lange Elektroden verwendet wurden.

## Abstract (en)

**Background:**

Hearing success in bimodally hearing patients with a cochlear implant (CI) and a hearing aid (HA) exhibits different results: while some benefit from bimodal CI and HA, others do not.

**Objective:**

The aim of this study was to investigate hearing success in terms of speech perception in bimodally fitted patients in relation to the cochlear coverage (CC) of the CI electrodes.

**Materials and methods:**

Using the OTOPLAN software (CAScination AG, Bern, Switzerland), CC was retrospectively measured from CT scans of the temporal bone of 39 patients, who were then categorized into two groups: CC ≤ 65% (CC^500^) and CC > 65% (CC^600^). Monaural speech intelligibility for monosyllables at a sound pressure level (SPL) of 65 dB in open field was assessed at various timepoints, preoperatively with HA and postoperatively with CI, and compared between the groups. In addition, speech intelligibility was correlated with CC in the entire cohort before surgery and during follow-up (FU).

**Results:**

Overall, no significant differences in speech intelligibility were found between CC^500^ and CC^600^ patients at any of the FU timepoints. However, both CC^500^ and CC^600^ patients showed a steady improvement in speech intelligibility after implantation. While CC^600^ patients tended to show an earlier improvement in speech intelligibility, CC^500^ patients tended to show a slower improvement during the first 3 months and a steeper learning curve thereafter. The two patient groups converged during FU, with no significant differences in speech intelligibility. There was no significant relationship between unimodal/unilateral free-field speech intelligibility and CC. However, patients with a CC of 70–75% achieved maximum speech intelligibility.

**Conclusion:**

Despite a nonsignificant correlation between CC and speech discrimination, patients seem to reach their maximum in unimodal/unilateral speech understanding mainly at 70–75% coverage. However, there is room for further investigation, as CC^500^ was associated with a shorter cochlear duct length (CDL), and long and very long electrodes were used in both groups.

## Physioanatomische Eigenschaften

Bei der Versorgung von Patienten mit einem Cochleaimplantat (CI) steht die individuelle Betreuung im Vordergrund. So berücksichtigt das behandelnde CI-Team z. B. die unterschiedlichen (patho)physioanatomischen Eigenschaften eines jeden Patienten, die von Innenohrmalformationen [36], Resthörerhalt [6, 16] über Prävention von Schwindel [29] bis zur großen Variabilität der Cochlealänge (Cochlear Duct Length, CDL) reichen [13, 33, 39]. Die verschiedenen CI-Hersteller bieten ein großes Portfolio von Elektroden unterschiedlicher Länge sowie auch unterschiedlicher Lage innerhalb der Cochlea [3, 10, 24, 27] an. Zudem sollte auch die Modalität bei der Versorgung beider Ohren berücksichtigt werden.

### Kategorien der Versorgungsmodalitäten

Ein Großteil der mit CI versorgten Patienten kann in 5 Kategorien unterteilt werden: 1. Einseitig taube Patienten (Single Sided Deafness, SSD), die auf einem Ohr normalhörend und auf dem anderen mit einem CI versorgt sind [1, 11, 41]. 2. Bimodal versorgte Patienten mit asymmetrischem Hören, die auf dem schlechter hörenden Ohr mit einem CI und auf dem besser hörenden Ohr mit einem Hörgerät (HG) versorgt sind, ohne dass hier eine CI-Indikation erreicht ist [1]. 3. Bimodal versorgte Patienten, die für beide Ohren eine Indikation für ein CI erreicht haben und mit 2 Modalitäten versorgt sind – auf dem schlechter hörenden Ohr mit einem CI und auf dem besser hörenden Ohr mit einem HG [9]. 4. Patienten mit elektrisch-akustischer Stimulation (EAS) am gleichen Ohr und HG am anderen Ohr. Diese Patienten haben ein gutes tieffrequentes Restgehör, sodass sie mit einem HG, das im CI-Audioprozessor integriert ist, mit gleichzeitiger elektrischer Stimulation über das CI selbst versorgt werden [16, 22]. 5. Beidseits ertaubte Patienten, die bilateral ein CI nutzen [31].

### Binaurale Integrationsvariabilität

Innerhalb dieser Kategorien wird jedoch eine große binaurale Integrationsvariabilität beobachtet. So geht für einige Patienten mit bilateraler Versorgung mit dem binauralen Hören ein essenzieller Benefit einher, während andere Patienten nur einen kleinen oder sogar keinen Vorteil erfahren. Auch Nachteile des binauralen Hörens, also binaurale Interferenz, ist möglich. Dieser Variabilität liegen verschiedene individuelle Charakteristika der Patienten zugrunde, die den Resthörerhalt [21], das Fehlen kortikaler Plastizität, die Ertaubungsdauer [5], die unterschiedliche Verarbeitungszeit von CI und HG [45, 46], eine Frequenzdiskrepanz zwischen CI- und HG-Ohr [4, 32] und Unterschiede in der automatischen Verstärkungsregelung (Automatic Gain Control) von CI und HG [40] umfassen. Bei einigen Patienten wird eine bimodale Interferenz beobachtet: Diese Patienten beschreiben ein besseres Hören bei der Nutzung nur eines Ohrs [28, 44]. Ein weiterer Parameter, der den Erfolg der bimodalen Versorgung möglicherweise beeinflusst, könnte die Elektrodenträgerabdeckung der Cochlea sein. Hier stellt sich die Frage, ob durch eine größere cochleäre Abdeckung (Cochlear Coverage, CC) monaural mit CI ein besseres tieffrequentes Hören erreicht wird und somit die Interferenzen mit der HG-versorgten Gegenseite vermieden werden können. In der vorliegenden Studie wird das monaurale Sprachverstehen mit dem CI bei bimodal versorgten Patienten in Abhängigkeit von der CC des CI-Elektrodenträgers untersucht.

## Material und Methoden

### Patientenkollektiv

Eine monozentrische retrospektive Analyse wurde bei insgesamt 39 bimodal versorgten Patienten durchgeführt, bei denen präoperative und postoperative audiometrische Daten und Röntgenaufnahmen nach Stenvers für die Lagekontrolle der CI-Elektroden (vollständige Insertion) zur Auswertung vorlagen. Die Patienten waren mit einer relativ langen FLEX28- (28 mm, aktive Stimulationslänge 23,1 mm) oder einer sehr langen FLEXSOFT-Elektrode (31,5 mm, aktive Stimulationslänge 26,4 mm) der Fa. MED-EL, Innsbruck, Österreich, versorgt [26].

### Cochleäre Abdeckung

Die Bestimmung der CC wurde bei allen Patienten mit der Software OTOPLAN (Fa. CAScination AG, Bern, Schweiz, Version 2) anhand der CT-Aufnahmen durchgeführt (CE-Zertifizierungsnummer: G1 17 10 95657 003). Die Software ermöglicht bei der präoperativen Planung anhand von DICOM®-Datensätzen (Digital Imaging and Communications in Medicine®) die Ausmessung der Cochlea, um z. B. die Insertionstiefe des CI-Elektrodenträgers oder die CC zu ermitteln [8].

Alle DICOM®-Datensätze wurden vor dem Hochladen in die Software von einem in Felsenbeinanatomie erfahrenen Radiologen zunächst auf die Qualität der Bildgebung und Malformationen des Felsenbeins untersucht. Ausschlusskriterien waren cochleäre Malformationen, CT-Schichtdicke ≥ 0,7 mm sowie Datensätze, die aus technischen Gründen nicht in die Software OTOPLAN übernommen werden konnten.

Anschließend wurde die Cochlea präoperativ mittels der Software in 3 Ebenen gemessen, die bereits in einer vorangegangenen Studie von Spiegel et al. ausführlicher beschrieben wurde [39]. Aus den ermittelten Werten „A-Wert“ (maximaler Abstand zwischen dem runden Fenster und der kontralateralen Wand), „B-Wert“ (Abstand zwischen den Wänden der Cochlea senkrecht zur Linie des A‑Werts) und „Höhe“ (Abstand senkrecht zur Basalwindung der Cochlea zum Apex) berechnet die Software anhand einer elliptisch-zirkularen Approximation (Elliptic-Circular Approximation, ECA) die Länge des Cochleagangs [35]. Zuletzt wird die CC anhand des zu erwartenden Insertionswinkels (Angular Insertion Depth, AID) aus dem präoperativen CT-Datensatz für die gewählte Elektrode rechnerisch bestimmt und die Frequenz-Ort-Zuordnung in der Cochlea mit der Greenwood-Funktion anhand der gewählten Elektrode geschätzt [35]. Eine CC von 100 % entspricht dabei 2,5 Windungen der Cochlea und ergibt somit eine AID von 900°. [14]. Alle beschriebenen Messungen wurden von 2 unabhängigen Untersuchern durchgeführt, die sowohl in Bezug auf die Messungen des anderen als auch auf die Elektroden verblindet waren, und die resultierenden Messergebnisse wurden gemittelt.

Zur weiteren Analyse der CC wurden die Probanden unabhängig vom Elektrodentyp in 2 etwa gleich große Vergleichsgruppen eingeteilt, eine mit einer CC von ≤ 65 % (mittlerer AID dieser Kohorte: 498,6°; als CC^500^ bezeichnet) und eine mit einer CC von > 65 % (mittlerer AID dieser Kohorte: 591,1°; als CC^600^ bezeichnet).

### Audiometrische Daten

Für die Durchführung der nach DIN EN ISO 8253 genormten Tonschwellenaudiometrie wurden Sinustöne nacheinander in verschiedenen Oktaven zwischen 250 Hz und 8 kHz in Luftleitung sowie Wobbeltöne im Freifeld in einem audiometrisch gedämmten Messraum präsentiert. Das nicht gemessene Gegenohr wurde bei entsprechender Indikation zusätzlich mit einem Rauschen gemäß Comité Consultatif International Télégraphique et Téléphonique (CCITT-Rauschen) vertäubt, um ein Überhören des nicht gemessenen Ohrs zu verhindern. In Luftleitung wurden die Töne über einen Kopfhörer ohne HG/CI dargeboten, im Freifeld dagegen über einen Lautsprecher, um die versorgte Hörschwelle mit HG/CI (Aufblähkurve) seitengetrennt zu ermitteln. Die daraus resultierenden gehörten präoperativen und postoperativen Werte in Dezibel Hörpegel (Decibel Hearing Level, dB HL), d. h. die unversorgten und versorgten Hörschwellen, wurden miteinander verglichen und beschrieben. Für die postoperativen Werte wurden die aktuellsten Daten zum Hörstatus aus der Patientenakte verwendet, die im Folgenden als Nachbeobachtungszeitpunkt (NBZ) bezeichnet werden.

Das Sprachverstehen, gemessen mit dem nach DIN 45621‑1 und DIN 45626‑1 genormten Freiburger Sprachtest bei 65 dB Schalldruckpegel (Sound Pressure Level, SPL) [15], wurde ebenfalls retrospektiv aus den elektronischen Patientenakten entnommen.

In dieser Studie wurde das monaurale Einsilbersprachverstehen im implantatversorgten Ohr präoperativ mit HG und postoperativ mit CI(‑Audioprozessor) untersucht. Die postoperativen Testzeitpunkte waren bei der Erstanpassung (EA) sowie einen Monat (1M), 3 Monate (3M) und ein Jahr (12M) nach Erstanpassung sowie zum NBZ.

### Statistische Analyse

Zur statistischen Auswertung der Daten wurden Microsoft Excel (Fa. Microsoft, Redmond, WA, USA, Version 2110) und das Statistical Package for Social Sciences (SPSS) Software (Fa. IBM, Armonk, NY, USA, Version 28) verwendet.

Der t‑Test für ungepaarte Stichproben wurde für normalverteilte Daten verwendet, um die Mittelwerte der CC und verschiedener Parameter der cochleären Morphologie (CDL, A‑Wert, B‑Wert und AID) sowie des Sprachverstehens zwischen den Gruppen CC^500^ und CC^600^ zu vergleichen. Für nicht normalverteilte Daten wurde der Mann-Whitney-U-Test verwendet, um die Medianwerte der cochleären Höhe und des Sprachverstehens zwischen den Gruppen CC^500^ und CC^600^ zu vergleichen. Zudem wurde der Zusammenhang zwischen Sprachverstehen für Einsilber und CC mithilfe des Pearson-Korrelationskoeffizienten untersucht. Das Signifikanzniveau betrug 0,05.

## Ergebnisse

### Demografie

Das Alter der 39 untersuchten Patienten betrug zum Zeitpunkt der Implantation im Median 65 Jahre (Minimum 15 Jahre, Maximum 90 Jahre). Bei 27 Patienten erfolgte die Implantation mit einer FLEX28- und bei 12 Patienten mit einer FLEXSOFT-Elektrode. Die Ätiologie der hochgradigen Schallempfindungsschwerhörigkeit bis hin zur Ertaubung wird in Tab. 1 dargestellt.

**Table 1:** 

Ätiologie	Anzahl *n*
M. Menière	6
Hörsturz	3
Vererbung	3
Intracochleäres Schwannom	2
Large-Vestibular-Aqueduct-Syndrom mit Mondini-Malformation	1
Unbekannt	24
*Gesamt*	39

### Cochleäre Abdeckung

Von den 39 Patienten wiesen 14 eine CC^500^ und 25 eine CC^600^ auf. Die cochleäre Abdeckung der CC^500^-Gruppe lag im Mittel bei 60,6 ± 3,6 %, die der CC^600^-Gruppe bei 73,1 ± 5,4 % und die der gesamten Kohorte bei 68,6 ± 7,7 %. Neben der CC sind weitere Parameter wie CDL, A‑ und B‑Wert, Höhe und AID in Tab. 2 aufgelistet. Die t‑Tests ergaben signifikante Unterschiede für CC (*t*(37) = −8,61; *p* < 0,001), CDL (*t*(37) = 3,67; *p* = 0,001), A‑Wert (*t*(37) = 2,74; *p* = 0,009), B‑Wert (*t*(37) = 3,77; *p* = 0,001), und AID (*t*(37) = −5,96; *p* < 0,001) zwischen der CC^500^- und der CC^600^-Gruppe. Nur die Höhe (*U* = 116,50; *Z* = −1,72; *p* > 0,05) unterschied sich nicht signifikant zwischen den beiden Gruppen.

**Table 2:** 

Morphologie	CC^500^ (*n* = 14)		CC^600^ (*n* = 25)		*p*-Wert	Gesamte Kohorte (*n* = 39)	
CC (%)	60,6	±3,6	73,1	±5,4	0,000^*^	68,6	±7,7
CDL (mm)	37,2	±1,4	35,0	±2,0	0,001^*^	35,8	±2,1
A‑Wert (mm)	9,6	±0,4	9,2	±0,4	0,009^*^	9,4	±0,4
B‑Wert (mm)	7,3	±0,3	6,7	±0,5	0,001^*^	6,9	±0,5
Höhe (mm)	4,3	±0,2	4,2	±0,4	0,324	4,2	±0,3
AID (°)	498,6	±29,5	591,1	±53,5	0,000^*^	557,9	±64,2

*AID* Angular Insertion Depth, errechnete Insertionstiefe; *CC* Cochlear Coverage, cochleäre Abdeckung; *CDL* Cochlear Duct Length, Länge des cochleären Gangs; *n* Anzahl; *SD* Standard Deviation, Standardabweichung

^* ^signifikante Werte

### Audiometrische Daten

**Figure 1:**
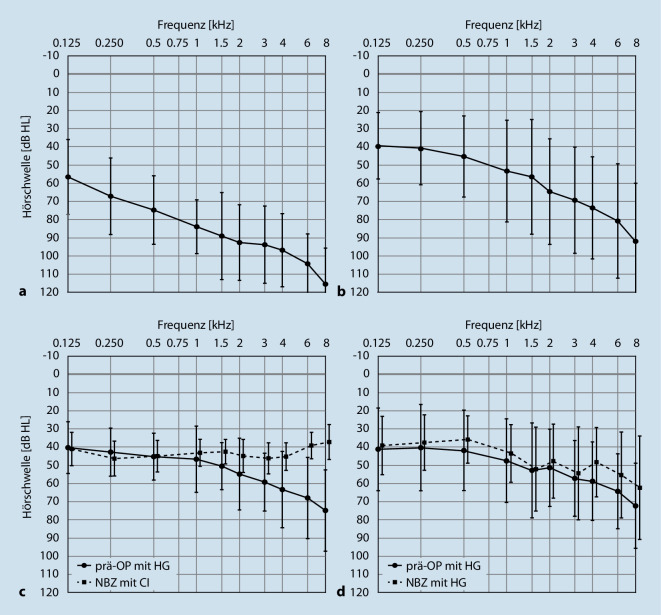

Die Tonaudiometrie in Luftleitung und im Freifeld wurde bei 39 bimodal versorgten Patienten durchgeführt. Dabei zeigt die Abb. 1a, dass die gemittelte Luftleitungshörschwelle präoperativ ohne HG für das implantatversorgte Ohr konstant von 57 dB HL bei 125 Hz bis hin zu 115 dB HL bei 8 kHz abfällt, bei einem Pure Tone Average, PTA, (0,5; 1; 2; 4 kHz) von 87,4 dB HL. Auch am nichtimplantatversorgten Gegenohr nahm präoperativ die gemittelte Luftleitungshörschwelle konstant von 39 dB HL bei 125 Hz bis hin zu 92 dB HL bei 8 kHz ab (Abb. 1b). Der PTA betrug dabei 59,3 dB HL.

Die versorgte Hörschwelle im Freifeld zeigte am implantatversorgten Ohr mit dem HG präoperativ ebenfalls einen abfallenden Verlauf von 40 dB HL bei 125 Hz auf 75 dB HL bei 8 kHz (PTA = 53,2 dB HL; Abb. 1c). Die Hörschwelle mit dem CI zum NBZ verläuft relativ konstant von 39 dB HL bei 125 Hz bis zu 37 dB HL bei 8 kHz (PTA = 44,7 dB HL; Abb. 1c). Der präoperative PTA für das mit HG versorgte Gegenohr betrug im Mittel 49,9 dB HL, der PTA zum NBZ im Mittel 43,9 dB HL (Abb. 1d). Weitere tonaudiometrische Daten vom implantatversorgten Ohr zum NBZ sind in Tab. 3 aufgeführt.

**Table 3:** 

	CC^500^ (*n* = 14)		CC^600^ (*n* = 25)		Gesamte Kohorte (*n* = 39)	
	Median	Wertebereich	Median	Wertebereich	Median	Wertebereich
PTA versorgte Hörschwelle (dB HL)	46,25	36,25–58,75	43,00	30,00–62,50	43,75	30,00–62,50
Sprachverstehen (%)	65	0–90	60	15–95	60	0–95

*CC *Cochlear Coverage, cochleäre Abdeckung;* dB* Dezibel; *HL* Hearing Level, Hörverlust; *PTA* Pure Tone Average, Reinton, der aus dem Durchschnitt für 0,5; 1; 2 und 4 kHz berechnet wurde; *Sprachverstehen *Sprachverstehen für Einsilber bei 65 dB SPL (Sound Pressure Level, Schalldruckpegel)

**Figure 2:**
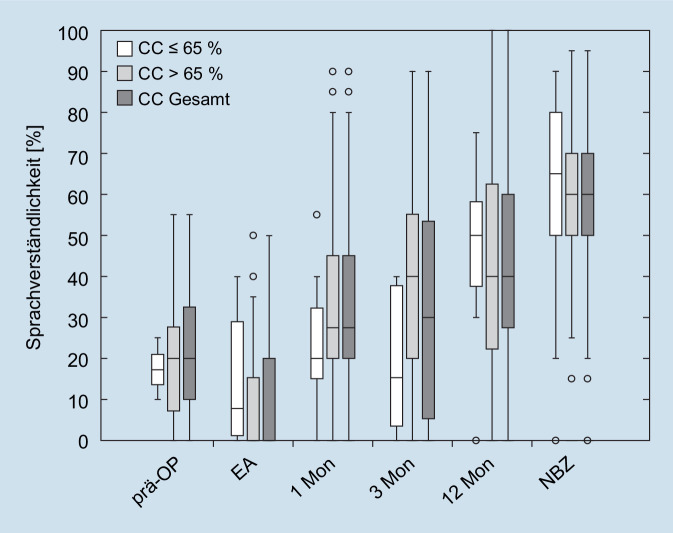

Im Test auf Normalverteilung waren alle Datensätze bis auf das Einsilberverstehen der C^600^-Gruppe zur EA und beim 1M-Termin normalverteilt (Shapiro-Wilk: *p* < 0,001 und *p* = 0,02). Die t‑Tests und Mann-Whitney-U-Tests ergaben keine signifikanten Unterschiede im Sprachverstehen zwischen CC^500^ und CC^600^ zu den untersuchten Beobachtungszeitpunkten. Die Box-Whisker-Plots in Abb. 2 zeigen einen Trend, dass sich das Sprachverstehen über die gesamte Kohorte für das implantatversorgte Ohr von 20 % des Gruppenmedians bei der präoperativen Messung auf 0 % des Gruppenmedians bei EA zunächst zu verschlechtern schien, sich aber postoperativ mit dem CI zu den folgenden Messzeitpunkten 1M nach EA (25 %), 3M (30 %), 12M (40 %) und zum NBZ (60 %) stetig zu verbessern schien. Bei der EA schienen die Patienten in der CC^500^-Gruppe bei der Sprachwahrnehmung zunächst besser abzuschneiden (7,5 %) als die Patienten in der CC^600^-Gruppe (0 %), allerdings ist dieser Unterschied nicht signifikant. Beim 1M-Termin schien sich jedoch eine leichte Verschlechterung der Sprachwahrnehmung bei den Probanden mit CC^500^ (20 %) im Vergleich zu den Probanden mit CC^600^ (27,5 %) zu zeigen. Nach 3M schienen die Probanden mit CC^500^ (15 %) ein deutlich schlechteres Sprachverstehen als die Probanden mit CC^600^ (40 %) aufzuweisen. Zwischen dem 3M- und dem 12M-Zeitpunkt schienen die Patienten mit CC^500^ (50 %) jedoch eine größere Verbesserung des Sprachverstehens zu zeigen als diejenigen mit CC^600^ (40 %). Beim NBZ schienen sich die beiden Gruppen im Sprachverstehen anzunähern. Weitere Daten zum Sprachverstehen mit dem implantatversorgten Ohr zum NBZ sind in Tab. 3 aufgeführt.

### Korrelation von cochleärer Abdeckung mit Sprachverstehen

**Figure 3:**
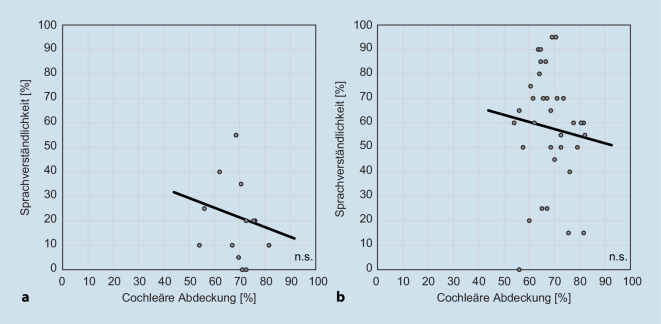

Eine Korrelationsanalyse ergab sowohl präoperativ monaural mit HG (*n* = 14; r = −0,16; *p* > 0,05) als auch postoperativ mit dem CI zum NBZ (*n* = 34; r = −0,09; *p* > 0,05) keine signifikante Korrelation zwischen Gesamt-CC (gesamte Kohorte aus CC^500^ und CC^500^) und Verständlichkeit bei 65 dB SPL. In Abb. 3 wird dargestellt, dass von Patienten mit einer CC von etwa 70–75 % ein maximales Sprachverstehen innerhalb der gesamten Kohorte erreicht wird.

## Diskussion

### Vorliegende Studie

Das Hauptziel dieser Studie war die Untersuchung eines Zusammenhangs zwischen der cochleären Abdeckung durch den Elektrodenträger bei bimodal mit CI und HG versorgten Patienten und dem monauralen Sprachverstehen für das implantatversorgte Ohr. Für die gesamte Patientengruppe konnte zu keinem Zeitpunkt eine signifikante Korrelation zwischen CC und monauralem Sprachverstehen bestimmt werden.

Im Gegensatz zu der Annahme der Autoren, dass eine vollständige Abdeckung der Cochlea durch den Elektrodenträger (CC = 100 %, entspricht einem AID von 900° bzw. einer Insertion von 2,5 Windungen) zu besserem Sprachverstehen führt, wurde in dieser Studie ein Maximum im Sprachverstehen bei etwa 70–75 % CC gefunden. Aufgrund der großen interindividuellen Variabilität des Sprachverstehens zu allen Beobachtungszeitpunkten, der geringen, nichtsignifikanten Korrelationskoeffizienten sowie der geringen Fallzahl sind die Ergebnisse jedoch nicht eindeutig interpretierbar. Um die Vermutung des Maximums bei 70–75 % CC bestätigen zu können, sind weitere Studien mit diesem Patientenkollektiv erforderlich. Auch Patienten mit 50–60 % CC sowie 80–90 % CC sollten in zukünftigen Studien berücksichtigt werden, da diese in der vorliegenden Studie unterrepräsentiert sind. Dadurch könnten weitere Einblicke in einen möglichen nichtlinearen Zusammenhang zwischen CC und Sprachverstehen gewonnen werden. Nicht näher untersucht wurde der Einfluss des präoperativen Resthörvermögens auf das Sprachverstehen der Probanden, d. h. ob Probanden mit Resthörvermögen grundsätzlich ein besseres Sprachverstehen erreichen als solche ohne Resthörvermögen und wie sich dies auf die entsprechende CC auswirkt. Insgesamt zeigten sich Unterschiede im Sprachverstehen innerhalb derselben Versuchspersonen zwischen den Testzeitpunkten, die u. a. auf psychobehaviorale Bedingungen wie Motivation oder Konzentration zurückzuführen sind. Diese Einflüsse erschweren eindeutige Interpretationen zusätzlich.

Dennoch zeigt die vorliegende Studie, dass sich die Gruppen CC^500^ und CC^600^ nach unterschiedlichem Sprachverstehen bei elektrischer Stimulation mittels CI – insbesondere die Gruppe CC^500^ benötigte anfänglich eine längere Lernphase im Sprachverstehen – mit ähnlichen Medianen und Streuungen sichtbar zum NBZ annähern.

### Vergleich mit anderen Studien

Bisherige Studien sind sich nicht einig, ob es einen Zusammenhang zwischen der cochleären Abdeckung durch den Elektrodenträger und dem monauralen Sprachverstehen mit CI gibt. So fanden Doubi et al. [12], die prälingual ertaubte Kinder unter 7 Jahren in 2 Gruppen aufteilten, 3 Jahre postoperativ keinen signifikanten Unterschied im Sprachverstehen mit CI, u. a. im Speech Intelligibility Rating Test, zwischen einer Gruppe mit CC < 85 % und einer mit CC ≥ 85 %. Sie schlossen daraus, dass eine Stimulation des apikalsten Bereichs der Cochlea keinen Vorteil für das Sprachverstehen bringt. Andere Studien haben eine mit der CC vergleichbare Metrik, die Insertionstiefe AID, verwendet und ihren Zusammenhang mit dem Sprachverstehen untersucht. Mehrheitlich wurde dabei über keine Korrelation zwischen den beiden Messgrößen berichtet [19, 20, 25, 37, 42, 43]. Ein gutes systematisches Review früherer Studien findet sich in der Arbeit von Heutink et al. [17], dabei wurde in 6 von 7 Studien keine signifikante Korrelation [19, 20, 25, 37, 42, 43] festgestellt. Andere Studien zeigten allerdings, dass die Insertionstiefe und das Sprachverstehen zusammenhängen. So berichteten O’Connel et al. [30] über eine signifikante positive Korrelation bei postlingual ertaubten Erwachsenen, gemessen 12–16 Monate postoperativ, mit einem Anstieg des englischsprachigen Consonant-Nucleus-Consonant-Score (CNC-Score) bei 60 dB(A) um 0,6 % pro 10° AID. Zu ähnlichen Ergebnissen mit positiver Korrelation kamen Canfarotta et al. [7] auf der Basis des CNC-Scores von unilateral mit CI versorgten Erwachsenen, gemessen 12 Monate nach der EA, und Heutink et al. [18] anhand des niederländischen Konsonant-Vokal-Konsonant-Tests, gemessen bei erwachsenen CI-Trägern mit mindestens einem Jahr unilateraler Hörerfahrung. Dem stehen Ergebnisse von Ketterer et al. [23] aus einer Studie gegenüber, in der erwachsene CI-Träger u. a. mit dem Freiburger Einsilbertest bei 65 dB SPL in regelmäßigen Abständen untersucht wurden; der Studie zufolge gab es Hinweise auf einen negativen signifikanten Zusammenhang, d. h. eine Abnahme des Sprachverstehens mit zunehmender Insertionstiefe … Obwohl in der vorliegenden Studie kein signifikanter Zusammenhang zwischen der Insertionstiefe und dem monauralen Sprachverstehen des implantatversorgten Ohrs gefunden wurden, scheint es hier einen negativen Einfluss größerer Insertionstiefen > 75 % auf das Sprachverstehen zu geben. Eine mögliche Erklärung dafür ist eine höhere Wahrscheinlichkeit, bei einer tieferen Insertion eventuell vorhandenes Restgehör zu schädigen. Ketterer et al. [23] führte ihre Ergebnisse auf windungsübergreifende Stimulation zurück, welche bei tief inserierten apikalen Elektroden auftreten kann. Allerdings wiesen in der Studie von Ketterer et al. [23] nur etwa 10 Ohren von 495 eingeschlossenen (etwa 2 %) eine CC von mehr als 75 % auf, womit diese tiefe Insertion eher unterrepräsentiert ist. Außerdem kann möglicherweise ein besseres präoperatives Sprachverstehen zu einem besseren postoperativen Sprachverstehen mit CI führen.

Ein Vorteil der elektrischen Stimulation im Apex der Cochlea ist die bessere Wahrnehmung von tiefen Tönen, was besonders bei der Musikempfindung ins Gewicht schlägt. Beim Hören von Musik erlebte eine gemischte Kohorte aus bilateralen und SSD-CI-Nutzern mit längeren Elektroden (31,5 mm) aufgrund der umfangreicheren apikalen Stimulation eine bessere Wahrnehmung der tieferen Frequenzen im Vergleich zu Nutzern mit 7,5 mm kürzeren Elektroden (24 mm) [34], was eine verbesserte Klangqualitätsdiskrimination (Sound Quality Discrimination) für Patienten mit langen Elektrodenträgern bedeutet.

Speck et al. [38] haben in einer Studie die Auswirkungen unterschiedlicher Elektrodenlängen (aktive Stimulationslänge: 15,0 mm vs. 19,1 mm vs. 23,1 mm) auf die Sprachverständlichkeitsschwellen (SVS) bei SSD-Patienten untersucht. Die SVS wurde in 2 verschiedenen Störgeräuschkonditionen untersucht: einmal mit Sprache und Störgeräusch von vorn (S0N0) und einmal mit Sprache auf der implantatversorgten Seite und Störgeräusch auf der normalhörenden Seite. In beiden Konditionen wurde kein signifikanter Unterschied in der SVS zwischen den Elektrodenträgerlängen festgestellt.

Gerade für bimodale CI-Nutzer, die auf der kontralateralen Seite ein HG tragen, sowie für einseitig ertaubte CI-Nutzer kann daher eine tiefe Insertion zu einem natürlicheren Hörempfinden sorgen, da die Elektroden der tieferen Frequenzen näher an die apikalen Spiralganglienzellen heranreichen, die nach Greenwoods Frequenz-Ort-Zuordnung in der Cochlea diesen tiefen Frequenzen entsprechen [14]. Dies ist vermutlich der Grund, warum in dieser Studie Patienten sich mit einer tieferen Insertion schneller an das Hören mit dem CI gewöhnen und dadurch den Lernprozess schneller durchlaufen als die Patienten mit einer geringeren Insertion, da das über das CI wahrgenommene Klangfeld in der Tonhöhe weniger verschoben ist. Ob dies für alle postlingual ertaubten Patienten der Fall ist, bleibt jedoch zu prüfen.

## Fazit für die Praxis

- In der vorliegenden Studie wurde kein signifikanter Zusammenhang zwischen dem monauralen Einsilbersprachverstehen unilateral mit dem Cochleaimplantat (CI) und der cochleären Abdeckung (CC) durch den CI-Elektrodenträger von bimodal (mit CI und Hörgerät, HG) versorgten Patienten festgestellt.
- Ein Trend war erkennbar: Das Sprachverstehen nimmt mit ansteigender CC zu bis zu einem Maximum bei etwa 70–75 % zu und fällt bei weiter ansteigender CC wieder ab.
- Der Grund für die Abwesenheit eines signifikanten Zusammenhangs kann die große Streuung in einem kleinen Patientenkollektiv und damit zu geringer statistischer Power der Studie sowie ein möglicher nichtlinearer Zusammenhang zwischen CC und Sprachverstehen sein, für dessen Analyse die lineare Pearson-Korrelation, auch teilweise bedingt durch die Kovariation des Elektrodenträgers (FLEX28, FLEXSOFT), nicht geeignet ist.
- Es wurde gezeigt, dass Patienten mit einer größeren Insertionstiefe schnellere Lernerfolge erzielen.
- Trotz unterschiedlicher CC^500^/CC^600^-Mittelwerte (59,2 ± 28,4 %/47,1 ± 21,9 %) und Mediane (65 %/60 %) war allerdings langfristig kein signifikanter Unterschied im Sprachverstehen ersichtlich.
- Für die Praxis erweist sich damit die präoperative Ausmessung der Cochlea und individualisierte Elektrodenauswahl als vorteilhaft, um alle Patienten mit einer optimalen individuellen Elektrodenlänge zu versorgen.
- Eine CC von 70–75 % zeigte sich in dieser Studie als guter Referenzpunkt für die anzustrebende cochleäre Abdeckung.
